# Lower risk of bloodstream infections for peripherally inserted central catheters compared to central venous catheters in critically ill patients

**DOI:** 10.1186/s13756-022-01180-1

**Published:** 2022-11-09

**Authors:** Vassiliki Pitiriga, John Bakalis, Kalliopi Theodoridou, Petros Kanellopoulos, George Saroglou, Athanasios Tsakris

**Affiliations:** 1 Department of Microbiology, Medical School, National and Kapodistrian University of Athens, 75 Mikras Asias Street, 11527 Athens, Greece; 2 Department of Internal Medicine, Metropolitan Hospital, 9 Ethnarchou Makariou Street, 18547 Athens, Greece

**Keywords:** Catheterization, Central venous catheter, Sepsis, Colonization, Bloodstream infection, Insertion site, Central line-associated bloodstream infection, Peripherally inserted central catheter

## Abstract

**Background:**

Peripherally inserted central venous catheters (PICCs) serve as an alternative to short-term central venous catheters (CVCs) for providing intravenous access in hospitalized patients. Although a number of studies suggest that PICCs are associated with a lower risk of central line-associated bloodstream infections (CLABSIs) than CVCs, recent data concerning specific patient groups support the contrary. In this regard, we are comparing CVC- and PICC-related CLABSI rates developed in a selected group of critically ill inpatients and evaluating the CLABSI microbiological distribution.

**Methods:**

The study was conducted at a tertiary care hospital in Greece between May 2017 and May 2019. We performed a two-year retrospective analysis of the data collected from medical records of consecutive adult patients who underwent PICC or CVC placement.

**Results:**

A total of 1187 CVCs placed for 9774 catheter-days and 639 PICCs placed for 11,110 catheter-days, were reported and analyzed during the study period. Among CVCs, a total of 59 (4.9%) CLABSIs were identified, while among PICCs, 18 (2.8%) cases presented CLABSI (*p* = 0.029). The CLABSI incidence rate per 1,000 catheter-days was 6.03 for CVC group and 1.62 for PICC group (*p* < 0.001). The CLABSI rate due to multidrug-resistant organisms (MDROs) among the two groups was 3.17 in CVC group and 0.36 in PICC group (*p* < 0.001). Within CLABSI-CVC group, the most common microorganism detected was MDR *Acinetobacter baumannii* (27.1%) followed by MDR *Klebsiella pneumoniae* (22%). In CLABSI-PICC group, the predominant microorganism was *Candida* spp. (33.3%) followed by non-MDR gram-negative pathogens (22.2%).

**Conclusions:**

PICC lines were associated with significantly lower CLABSI rates comparing to CVC although they were in place longer than CVC lines. Given their longer time to the development of infection, PICCs may be a safer alternative for prolonged inpatient IV access. The high prevalence of CLABSI-MDROs depicts the local microbial ecology, emphasizing the need of public health awareness.

## Introduction

Central venous catheters (CVCs) are widely used in clinical setting due to their functional role in intravenous therapy, laboratory testing, and hemodynamic monitoring [1]. While they are often placed for saving patients’ lives, they are closely associated with considerable morbidity and mortality [2] as well as an increased risk of mechanical complications and infections [3]. Despite the implementation of CVC hygiene bundles, central line-associated bloodstream infections (CLABSIs) remain an important healthcare-associated complication [4] that can adversely affect patient care and is still strongly related to increased mortality rates [5].

In previous years [6], peripherally inserted central venous catheters (PICCs) were introduced into the treatment of hospitalized patients as a safer alternative to the conventional type of CVCs, particularly for patients to whom long-term venous access is required, and has gained increasing acceptance. Due to the various advantages they exert, at present, PICCs are the most common central venous catheters used in inpatient settings since they have been reported to reduce the incidence of CLABSIs not only in hospitalized patients but also in outpatients receiving intravenous medication at home compared with traditional CVCs [7–12].

However, recent studies [8, 9] indicate that PICC-related CLABSIs rates vary among different patient groups and in some cases they are actually similar or even higher than of CVCs related CLABSIs rates [10]. PICC-related bloodstream infection represents a serious complication that may contribute to increased costs and mortality rates [11]. As the use of PICCs expands to include vulnerable populations, such as critically ill patients, determining the risk of CLABSI posed by PICCs relative to CVCs in this specific group is crucial and needs further attention. In this regard, we conducted the present study in order to compare CVC- and PICC-related CLABSI rates developed in critically ill hospitalized patients. We also aimed to analyse and compare the CLABSI pathogen distribution in each group of events.

## Materials and methods

We performed a retrospective analysis of data collected from the medical records of consecutive adult hospitalized patients who underwent PICC and CVC placement at the Metropolitan Hospital, Piraeus, Greece, between May 2017 and May 2019. Metropolitan Hospital is a large tertiary-care hospital that includes several surgical, oncological and internal medicine units as well as a general intensive care unit (ICU). The observational study was approved by the institutional review board.

### Data collection

After insertion, catheters were checked using a check-box form containing the patient’s diagnosis, operator’s name, site chosen, date placed and removed, date of intensive care units (ICU) discharge or death, mechanical ventilation, arterial catheters, parenteral nutrition, and daily clinical assessment (e.g., discharge, erythema, and tenderness) of possible catheter infection. The operator inserting the catheter entered the initial data; nurse personnel entered data the following days while the infection control nurse monitored data collection 3–4 times per week. Data was retrospectively collected from two different data sources: (1) medical database (for demographic and clinical data related to the patient’s admission and clinical course) and (2) Clinical Laboratory and hospital infection control team database (for blood culture and antibiotic susceptibility results).

### CVC and PICC insertion protocol

In our hospital triple lumen, non-antibiotic impregnated catheters (Arrow model, total provided by Arrow®/Teleflex®, Wayne, USA) are mainly used. Double lumen catheters (Arrow®/Teleflex®, Wayne, USA), are also used but in a lower percentage, particularly in patients that do not require complex therapeutic interventions. The choice of the site of insertion was left to the discretion of the physician caring for the patient. Maximal sterile barrier precautions (large sterile drape; surgical hand antisepsis; and mask, cap, sterile gloves, and gown) were used at catheter insertion according to CDC recommendations.

### Catheter care protocol

Standardized CVC/PICC care practices were implemented by a highly proficient nursing staff. Every couple of days or earlier if clinically required, the nursing staff changed the dressing, cleaned the skin site and the catheter hub with iodine solution, and changed the intravenous accessory tubing. Catheters were removed when (a) there was evidence or suspicion of infection, (b) when the catheter was no longer required.

### Culture techniques

All catheters were examined for the presence of pathogens either as a routine after removal or after suspicion of infection. After disinfecting skin around the catheter entry site, the proximal 4–5 cm part of the tip was cut off using sterile scissors. The specimen was placed in a sterile container and transported to the department of microbiology within 15 min at room temperature. The intradermal and intravascular portion of the catheter was analyzed by the semiquantitative culture technique described by Maki et al. [12]. According to Maki’s technique, catheter-tip culture is considered positive in the presence of ≥ 5 colony-forming units (CFU) growth of any organism.

Blood cultures were incubated in Becton Dickinson Bactec (BD Bio-sciences, USA) in aerobic and anaerobic broth media. Identification of isolates and antimicrobial resistance patterns were determined by the VITEK®2Automated Compact System (BioMérieux Co., France). E-test (BioMérieux Co., France) was performed as an additional test, in order to confirm the resistance phenotypes reported by the VITEK System, according to the standard laboratory procedures.

### Definitions

*CVC* was defined as any central venous access device inserted into the internal jugular, subclavian, or femoral vein that terminated in the inferior vena cava or right atrium.

*PICCs* were defined as catheters inserted in the basilic, cephalic, or brachial veins of the upper extremities with tips that terminated in the superior vena cava or right atrium.

Catheter infection definition were based on the Centers for Disease Control bloodstream infection guidelines and the semi-quantitative culture technique by Maki et al.

*Catheter associated BSI (CLABSI)* was defined as a laboratory confirmed BSI (a positive blood culture with no other apparent source of infection) occurring in the presence of a CVC/PICC or within 48 h of catheter removal.

*Catheter-days* was defined as the number of CVCs/PICCs presents among all units’ patients at 08:00 h each morning.

*Multidrug-resistant organisms (MDROs)* were defined as **s**pecies of microorganisms that exhibit antimicrobial resistance to at least one antimicrobial drug in three or more antimicrobial categories. This definition concerns both gram-positive and gram-negative bacteria [13].

### Statistical analysis

Descriptive analysis to characterize patients’ population were reported as count (percent) or mean value (± standard deviation) for qualitative and quantitative variables, respectively, and were compared between the two groups using Chi-square test or Student’s *t* test, as appropriate. A two-sided *P* value of ≤ 0.05 was considered as statistically significant.

## Results

A total of 1187 CVCs for 9774 catheter-days and 639 PICCs for 11,110 catheter-days were placed and analyzed during the two-year study period. Among CVCs, a total of 59 (4.9%) CLABSI cases with clinical symptoms were identified. Among PICCs, 18 (2.8%) cases presented CLABSI during our survey. Patients’ demographic characteristics and hospitalization data that was considered as baseline indicators of illness severity are presented in Table 1. No statistically significant differences were observed among the two groups. The majority of the study population was catheterized with three-lumen catheters. Two-lumen catheters were placed in only 2 cases (2.5%). Patients who were discharged with PICCs and CVCs and patients with catheters urgently placed were not excluded from the study. The proportion of these incidences were relatively low, of approximately 5% and 1%, respectively.

**Table 1:** Demographical characteristics and indicators of illness severity among CVC/PICC groups

Characteristics	CVC-patients (*n* = 59)	PICC-patients (*n* = 18)
	N (%)	N (%)
*Demographics*		
Age, mean ± SD (years)	55.08 ± 19.8	60.24 ± 17.5
Gender (M/F)	41/18	13/6
Obesity	19 (32.2)	9 (50)
*Indicators of illness severity*		
ICU admission	32 (54.2)	9 (50)
Total parenteral nutrition	38 (63.8)	9 (50)
APACHE II at inclusion, (mean ± SD)	12.8 ± 8.2	10.2 ± 6.5
Length of hospital stay before IV catheter, (mean ± SD)	57.7 ± 35.8	41.6 ± 19.5
Duration of catheter use, (mean ± SD)	16.19 ± 10.1	28.4 ± 12.5
In-hospital mortality	19 (32.2)	6 (33.3)

IV, Intravenous; SD, standard deviation; M/F, male/female

The mean duration of CVCs placement was 16.2 ± 10.1 days (Range: 2–56 days), while the mean duration of PICCs placement was 28.4 ± 12.5 days (Range: 2–93 days). The CLABSI incidence rate per 1000 catheter-days was 6.03 for CVC group and 1.62 for PICC group (*t*-test, *p* < 0.001). The median time to development of infection was 23 days in the patients with a PICC and 13 days in patients with CVC (*t*-test, *p* = 0.03).

The CLABSI rate due to multidrug-resistant organisms (MDROs) among the two groups was 3.17 in CVC group and 0.36 in PICC group (*t*-test, *p* < 0.001) (Table 2). The distribution of pathogens in CLABSI-CVC and CLABSI-PICC groups are presented in Table 3. Within CLABSI CVC group, the most common microorganism isolated was MDR *Acinetobacter baumannii* (27.1%) followed by MDR *Klebsiella pneumoniae* (22%). In CLABSI-PICC group, the predominant microorganism isolated was *Candida* spp. (33.3%) followed by non-MDR gram-negative pathogens (22.2%).

**Table 2:** CLABSI and CLABSI-MDROs incidence rate among CVC/PICC groups

	PICCs	CVCs	*P*-value
No of catheters	639	1187	
Total catheter-days	11,110	9774	
No of CLABSI	18	59	X^2^ = 4.74 *P* = 0.029
CLABSI incidence rate (per 1000 cath-days)	1.62	6.03	*T*-test, *p* < 0.001
No of CLABSI-MDRO	4	31	X^2^ = 8.7 *P* = 0.003
CLABSI-MDRO incidence rate (per 1000 cath-days)	0.36	3.17	*T*-test, *p* < 0.001

**Table 3:** Pathogen distribution among CVC/PICC groups

Pathogens	CVC-CLABSIs (n = 59)	PICC-CLABSIs (n = 18)
	N (% of total isolates)	N (% of total isolates)
*Gram-negative bacteria*		
MDR *K. pneumoniae*	13 (22)	2 (11.1)
MDR *A. baumannii*	16 (27.1)	1 (5.6)
MDR *P. aeruginosa*	2 (3.4)	1 (5.6)
Non-MDR *P. mirabilis*	1 (1.7)	–
Non-MDR *P. aeruginosa*	1 (1.7)	–
*Gram-positive bacteria*		
Coagulase-negative staphylococci	9 (15.3)	3 (16.6)
Methicillin-resistant *S. aureus*	3 (5.0)	–
*Enterococcus* spp*.*	2 (3.4)	1 (5.6)
*Yeasts*		
*Candida* spp.	6 (10.2)	6 (33.3)
Other bacterial pathogens	6 (10.2)	4 (22.2)

## Discussion

Since central lines are increasingly prevalent in critically-ill patients, it is important to take into serious consideration that CLABSI is a leading cause of preventable healthcare-associated infections leading to longer hospital stays, higher hospital costs, and significant mortality [14].

Our findings support that PICC utilization carries less risk for the development of CLABSIs than CVCs since PICC-CLABSI rates were considerably lower compared to CVC-CLABSIs among the study group. Furthermore, in patients with PICC-CLABSIs, the mean time to infection was 12 days longer than the one of patients with CVC-CLABSIs, suggesting the use of PICCs as an attractive alternative to CVC lines, especially in the group of critically-ill patients or those requiring prolonged inpatient IV access.

There have been several reports regarding comparison of the rate of CLABSI associated with PICCs and CVCs in hospitalized patients in different medical care units, however their outcomes are widely conflicting. Furthermore, limited data exist on the CLABSI risk comparison between PICCs and CVCs events in critically-ill patients combined with the pathogen epidemiology of each category. This low CLABSI rate associated with PICCs is in line with the prevailing opinion [15, 16] that supports the extent use of PICCs in daily practice due to their less invasive insertion technique, the low rate of mechanical complications, and their safety due to the lower infectious rates and the easy removal techniques.

With respect to infections, PICCs have been acknowledged to be safer than other central lines by many authors, possibly due to minor microbial density and lower temperature of the PICC placement site compared to those of other central venous catheters which include neck and groin [17].

In our study, A*cinetobacter* spp*.*, *K. pneumoniae,* coagulase-negative staphylococci and *Candida albicans* were the most common microorganisms isolated from CVCs. Based on the international reports on CLABSI pathogen epidemiology available from numerous studies, causative microorganisms typically originate from the normal resident flora of the skin present at the insertion site, which are mostly consisting by gram-positives such as coagulase-negative staphylococci*,* and *Corynebacterium* spp [18–20]. However, previous studies report a change in CLABSI pathogen profile, with Gram-negatives either predominating in the panel of isolated organisms or displaying increasing trends [21–23]. This change in the types of pathogens could be attributed to the broad implementation of infection control programs targeting Gram-positive organisms [24]. In our study the epidemiology profile of CVC-CLABSI pathogens reflects the Greek ICU pathogen profile depicted by previous reports, where MDR *A. baumannii* is frequently isolated [25]. This emergence of MDROs has created a great concern on medical settings in Greek hospitals, especially for ICU patients [26]. Based on the annual data of antimicrobial resistance rates reported by our hospital’s clinical laboratory, the rate of the three most commonly isolated MDR Gram-negatives (*A. baumannii, K. pneumoniae, P. aeruginosa*) recovered from hospitalized patients was > 20% during the study period, mainly originated from ICU patients. Moreover, given the fact that most CVC-CLABSI patients in our study population had a prolonged stay in ICU and had been exposed to many different classes of antibiotics, it is not surprising that MDR Gram-negatives caused the majority of CLABSIs in our setting.

In contrast, the pathogen distribution in PICC CLABSIs exhibited a different bacterial profile, with *Candida * spp. being the predominant isolated microorganism. This is reasonable since the duration of catheter utilization before the development of infection was significantly longer in patients with PICCs compared with those with CVCs. Indeed, among many risk factors, prolonged use of catheters placement consists an independent risk factor for the development of invasive candidemia in hospitalized patients [27, 28]. Our findings are in agreement to other studies [29] supporting that the presence of a peripherally inserted central catheter significantly correlates with candidemia. *Candida* species have been reported as the fourth most common causes of bloodstream infections (BSIs) among immunocompromised inpatients worldwide in the last two decades [30, 31].

This study has potential limitations, since the development of CLABSI in patients with PICCs vs CVCs is known to be influenced by a combination of different factors of both patients and devices' characteristics. Therefore, the present retrospective analysis could be subject to considerable biases and confounding, mainly regarding the severity of illness and the different duration of catheterization between the two study-populations. Thus, it would be important to examine the characteristics of the whole population that was catheterized with PICCs and CVCs during that period, in order to better understand the difference in patient risk between the two groups. Unfortunately, we have had limited access to data of the entire population. Based on available data from almost 30% of the whole catheterized population (347 patients with CVCs and 175 with PICCs), no significant differences in APACHE score and length of hospital stay before catheterization were determined. This was considered reasonable since PICCs and CVCs are applied to the same patient population in our hospital, that include severe cases after surgical operation, septic and multi-trauma patients. Moreover, considering the demographical characteristics of both groups that developed CLABSIs, no significant differences in underlying diseases or medical history were observed. In addition, the APACHE score, which is considered as the most straightforward single variables to estimate severity of illness, was similar between the above two groups (Table 1). Regarding the different time duration of catheterization for PICCs and CVCs, our previous study have shown that longer duration of catheterization is linked with greater rates of CLABSIs [32]. In the current study, though time duration of PICCs’ catheterization was considerably longer than of CVCs’, the rates of PICCs CLABSIs were significantly lower than those of CVCs.

## Conclusions

The findings of the present study support the beneficial use of PICC lines compared to CVCs in critically ill patients, in terms of CLABSI rates, despite the longer indwelling time of PICCs. Moreover, a substantial swift in the epidemiological profile of CLABSIs pathogens towards a high proportion of Gram-negative pathogens and specifically MDROs was noted in CVC-CLABSIs.

## Data Availability

All data generated or analyzed during this study are included in this published article.

## Abbreviations

CLABSI: Central-line associated bloodstream infection
CVC: Central venous catheter
ICU: Intensive care unit
MDRO: Multidrug-resistant organism
PICC: Peripherally inserted central venous catheter
